# The outbreak of COVID-19 in Taiwan in late spring 2021: combinations of specific weather conditions and related factors

**DOI:** 10.1007/s11356-021-17055-8

**Published:** 2021-10-20

**Authors:** Shih-An Chang, Chia-Hsuan Kuan, Chi-Yen Hung, Tai-Chi Chen Wang, Yu-Sheng Chen

**Affiliations:** 1 Department of Chinese Acupuncture and Traumatology, Center of Traditional Chinese Medicine, Chang Gung Memorial Hospital, Taoyuan, Taiwan; 2 Department of Atmospheric Sciences, National Central University, Taoyuan, Taiwan; 3 Graduate Institute of Clinical Medical Sciences, Chang Gung University, Taoyuan, Taiwan; 4 Taiwan Huangdi‑Neijing Medical Practice Association (THMPA), Taoyuan, Taiwan

**Keywords:** COVID-19, Weather, Meteorological parameters, “Unease environmental condition factor (UECF)”, “Major-Yin-control year”

## Abstract

This study aimed to investigate the impact of weather conditions on the daily incidence of the COVID-19 pandemic in late spring 2021 in Taiwan, which is unlike the weather conditions of the COVID-19 outbreak in 2020. Meteorological parameters such as maximum daily temperature, relative humidity, and wind speed were included. The Spearman rank correlation test was used to evaluate the relationship between weather and daily domestic COVID-19 cases. The maximum daily temperature had a positively significant correlation with daily new COVID-19 cases within a 14-day lag period, while the relative humidity and wind speed has a fairly high correlation with the number of daily cases within a 13- and 14-day lag, respectively. In addition, the weather characteristics during this period were an increasingly high temperature, with steady high relative humidity and slightly decreasing wind speed. Our study revealed the weather conditions at the time of the domestic outbreak of COVID-19 in Taiwan in May 2021 and the possible association between weather factors and the COVID-19 pandemic. Further large-scale analysis of weather factors is essential for understanding the impact of weather on the spread of infectious diseases.

## Introduction

Large-scale outbreaks of coronavirus disease 2019 (COVID-19) caused by infection with severe acute respiratory syndrome coronavirus 2 (SARS-CoV-2) led to high fatality rates in many countries around the world after the first case was reported in December 2019 (WHO 2020). In Taiwan, the first case of SARS-CoV-2 infection was confirmed on January 21, 2020 (Cheng et al. 2020a). Despite widespread COVID-19 cases around the world in last years, the spread of COVID-19 in Taiwan was effectively controlled with quick border quarantine and precise case investigation due to the experience obtained from the spread of severe acute respiratory syndrome (SARS) in 2003; these efforts resulted in a relatively low number of confirmed COVID-19 cases in 2020 (Cheng et al. 2020b; Lin et al. 2020; Wang et al. 2020). Nevertheless, another wave of COVID-19 outbreaks caused by the SARS-CoV-2 alpha variant in Taiwan was reported in May 2021, with a resurgent increase in the number of new COVID-19 cases (Hannah Ritchie 2020).

Previous studies have investigated the relationship between weather conditions and widespread outbreaks of COVID-19 in different regions with various meteorological parameters (including temperature, humidity, and wind speed) during the past year (Pani et al. 2020; Şahin 2020; Tosepu et al. 2020). Despite the diverse results, most studies concluded a negative correlation between temperature and COVID-19 transmission (McClymont & Hu 2021, Paraskevis et al. 2021). Laboratory research found that the structural stability of the SARS-CoV-2 virus quickly decreases at warm temperatures (Sharma et al. 2021). It was believed that the incidence of COVID-19 can drop in the summertime. Nevertheless, a massive outbreak of COVID-19 at high ambient temperatures began in Taiwan this time. Hence, our study aimed to investigate the impact of weather conditions on the daily incidence of the COVID-19 pandemic in late spring 2021 in Taiwan.

## Methods

### Study area

Banqiao, a district of New Taipei City, lies between 25° 00′ north latitude and 21° 26′ east longitude. The nearest meteorological observation station was close to Wanhua District, Taipei, which was the site of the domestic outbreak of COVID-19 in May 2021.

### Data collection

The data of daily domestic COVID-19 cases of Taiwan for the period of May 1–May 28, 2021 were obtained from Taiwan Centers for Disease Control. May is defined as “late spring” in the Northern Hemisphere and the change of monsoon in Taiwan (Jan et al. 2002; Trenberth 1983). The weather data for the period of May 2021 were gathered from the Central Weather Bureau, Taiwan. The data consist of maximum daily temperature (°C), humidity (%), and wind speed (m/s).

### Data analysis

The Spearman rank correlation test, a non-parametric test to determine the strength and direction of association between two variables, was used to evaluate the relationship between weather and daily domestic COVID-19 cases because of the non-normal distribution of the data.

## Results

**Fig. 1:**
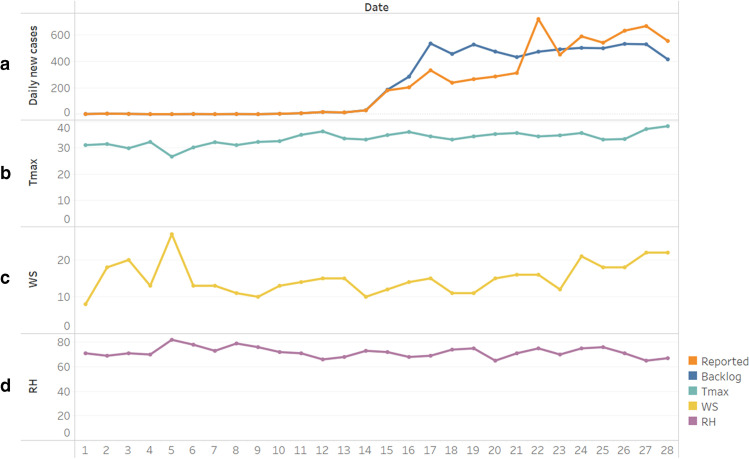
**(a)** The daily domestic COVID-19 cases of Taiwan for the period of May 1- May 28. The daily variations in different meteorological parameters including **(b)** *T*_max_, maximum daily temperature (°C), **(c)** WS, wind speed (m/s), and **(d)** RH, relative humidity (%) in Banqiao

The daily domestic COVID-19 cases of Taiwan for the period of May 1–28 are shown in Fig. 1(a). Due to a large amount of PCR tests and the delay of the results report, the Central Epidemic Command Center (CECC) used the backlog to retrospectively adjust the daily newly confirmed case to reflect the true extent of the outbreak. Hence, both daily reported and backlog local cases are included but analyzed separately. Figure 1(b–d) shows the daily variations in different meteorological parameters, including maximum daily temperature (°C), humidity (%), and wind speed (m/s), at the Banqiao meteorological observation station, and their relationship with the daily reported cases.

Table 1 presents the results of Spearman’s correlation analysis between the new daily domestic COVID-19 cases (including both reported and backlog) and variable meteorological parameters. The maximum daily temperature was significantly correlated with daily reported domestic COVID-19 cases (*p* < 0.01, *r* = 0.718), and a similar trend was also observed on the backlog cases (*p* < 0.01, *r* = 0.658). The wind speed was only significantly correlated with daily reported domestic COVID-19 cases (*p* < 0.05, *r* = 0.420) but not with the backlog. The relative humidity was not significantly correlated with daily cases.

**Table 1:** Spearman’s correlation coefficient between the new daily domestic COVID-19 cases (including both reported and backlog) and variable meteorological parameters

Meteorological parameters	Daily domestic COVID-19 cases (reported)	Daily domestic COVID-19 cases (backlog)
Maximum daily temperature	0.718**	0.658**
Wind speed	0.420*	0.301
Relative humidity	− 0.289	− 0.284

^*^Correlation is significant at the 0.05 level; ^**^correlation is significant at the 0.01 level

**Fig. 2:**
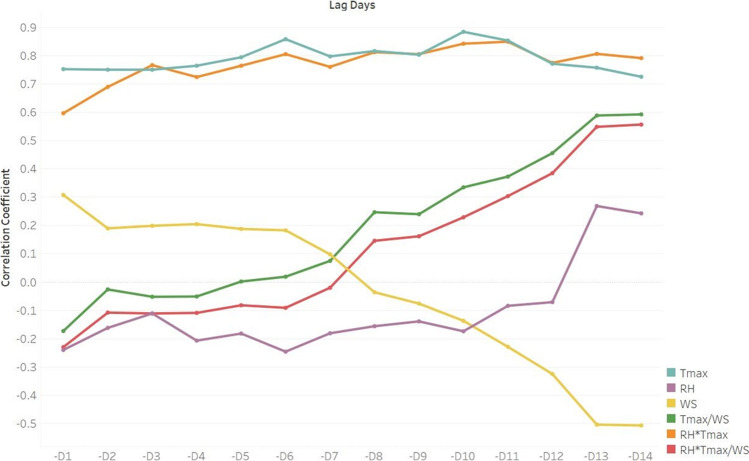
The correlation coefficient of maximum daily temperature, wind speed, relative humidity, and the accompanying effect of these parameters with the new daily cases within 14 days

In addition, considering that the incubation period of SARS-CoV-2 varies from 1 to 14 days and considering the lag effects of weather conditions on daily new cases, we chose the maximum temperature and wind speed for further evaluation. Table 2 demonstrates the correlation between these two parameters within a 14-day lag (*D*0 = the weather on the day, − *D*1 = the weather one day ago) and the number of daily reported domestic COVID-19 cases. The results revealed a high correlation between the maximum temperature within a 14-day lag and the daily reported domestic COVID-19 cases (*p* < 0.01), while wind speed over 14 days had the highest correlation with the number of daily cases. To consider the accompanying effects of these parameters together with their correlation with the number of daily cases, the maximum temperature divided by wind speed (*T*_max_/*WS*), maximum temperature multiplied by relative humidity (*T*_max_ × *RH*), and all factors considered together (*T*_max_ × *RH*/*WS*) were applied to evaluate the correlation with the number of daily cases within 14 days; the results are shown in Fig. 2.

**Table 2:** Spearman's correlation coefficient between the new daily domestic COVID-19 cases (including both reported and backlog) and variable meteorological parameters within a 14-day lag

Lag days	Temperature maximum		Wind speed		Relative humidity	
Daily case	Reported	Backlog	Reported	Backlog	Reported	Backlog
On the day (D0)	0.718**	0.658**	0.42*	0.301	− 0.289	− 0.284
1 day ago (− D1)	0.753**	0.69**	0.308	0.195	− 0.24	− 0.172
2 day ago (− D2)	0.751**	0.762**	0.19	0.131	− 0.162	− 0.143
3 day ago (− D3)	0.751**	0.783**	0.199	0.157	− 0.111	− 0.222
4 day ago (− D4)	0.765**	0.76**	0.205	0.185	− 0.207	− 0.281
5 day ago (− D5)	0.795**	0.802**	0.188	0.164	− 0.182	− 0.164
6 day ago (− D6)	0.859**	0.84**	0.183	0.172	− 0.246	− 0.299
7 day ago (− D7)	0.798**	0.785**	0.098	0.109	− 0.181	− 0.217
8 day ago (− D8)	0.817**	0.802**	− 0.036	− 0.047	− 0.156	− 0.086
9 day ago (− D9)	0.804**	0.743**	− 0.076	− 0.107	− 0.139	− 0.067
10 day ago (− D10)	0.885**	0.814**	− 0.137	− 0.179	− 0.174	− 0.092
11 day ago (− D11)	0.854**	0.754**	− 0.229	− 0.293	− 0.084	0.05
12 day ago (− D12)	0.772**	0.656**	− 0.325*	− 0.295	− 0.071	0.18
13 day ago (− D13)	0.758**	0.696**	− 0.504**	− 0.447**	0.269	0.246
14 day ago (− D14)	0.726**	0.640**	− 0.507**	− 0.402*	0.243	0.213

^*^Correlation is significant at the 0.05 level; ^**^correlation is significant at the 0.01 level

## Discussion

As the first known imported case of COVID-19 in Taiwan was confirmed on January 21, 2020, a series of policy decisions and social norms were subsequently announced by the Taiwanese government and Taiwan Centers for Disease Control (Taiwan CDC) (Lin et al. 2020). With the lessons learned from the 2003 SARS outbreak, the rapid implementation of disease prevention made the COVID-19 pandemic of Taiwan effectively controlled in 2020 (Cheng et al. 2020a). However, the resurgence of coronavirus cases was found in May 2021 with local rapid spread, forcing the government to upgrade the disease control policies. Although loosening border control and quarantine and the relatively very low vaccination rate might be one of the reasons why the new outbreak happened (Anderson et al. 2020; Chen et al. 2020), the second spike of daily COVID-19 cases in spring 2021 was also observed in North America and Europe, with more people receiving the COVID-19 vaccine since December 2020 (WHO 2020). Hence, we suppose that some other global or regional factors, such as weather variables, may also play an important role in COVID-19 transmission at this time.

After a year of analyses and research on the impact of weather variables on the COVID-19 pandemic in 2020, heterogeneous results have been reported, but most of the studies showed a negative correlation between temperature and COVID-19 incidence (McClymont & Hu 2021, Paraskevis et al. 2021). Another study revealed that both temperature and relative humidity were negatively related to the daily new cases of COVID-19 worldwide (Wu et al. 2020), indicating that the effect of weather conditions on the spread of COVID-19 may be global. Laboratory studies have reported that SARS-CoV-2 is sensitive to high temperature and humidity; it loses viability rapidly with increasing temperature (Chan et al. 2020; Chin et al. 2020). Most of the study results suggested that the growth trend of COVID-19 may slow with an increase in temperature and humidity when the Northern Hemisphere enters summer (Dzien et al. 2020, Sagripanti & Lytle 2020).

**Fig. 3:**
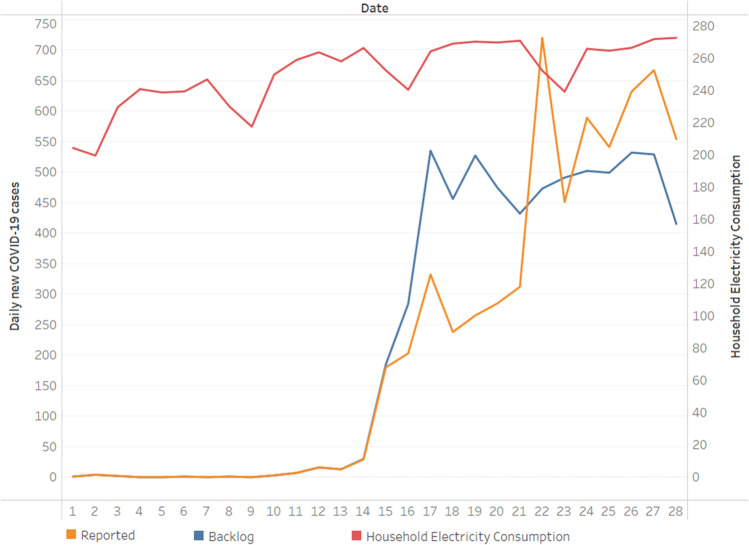
Daily household electricity consumption of Taiwan for the period of May 1–May 28 and its relationship to daily domestic COVID-19 cases

Taiwan belongs to tropical and subtropical climate zones, with warm temperatures in late spring. The outbreak of COVID-19 in the spring of 2021 occurred under high temperature, which is different from the spread in winter or early spring of the past year. We used the weather data in late spring with the period of May 1–May 28 for analysis. During this period, the Pacific high pressure system unusually persisted and affected the meteorological parameters with high temperature. The plum rain, a climatic phenomenon in East Asia caused by monsoon change and stationary fronts which often brings heavy rainfall (Chen 1983), also arrived lately until May 29, 2021. From the analysis, both the temperature average and maximum temperature were significantly positively correlated with daily new COVID-19 cases (see Table 1). Although there was no significant correlation with the relative humidity and the number of cases since Taiwan is an island surrounded by sea with a comparatively high relative humidity within limited variation the entire year, the characteristics during this period were increasingly high temperature, with steady high relative humidity and slightly decreasing wind speed (see Fig. 1). Since the environmental condition is one of the factors affecting the COVID-19 pandemic and has been proven to affect the spread by many studies (Hu et al. 2021; Rahman et al. 2020), and the weather in Taiwan in May 2021 is unusually hot and uncomfortable, we propose that the weather factors mentioned above caused people to feel sick and be more vulnerable to becoming ill, and accordingly named them “unease environmental condition factor (UECF).” Under the UECF, people tend to gather indoors with air-conditioning, and there is a significantly positive correlation between the daily confirmed COVID-19 case and household electricity consumption in Taiwan during this period (*p* < 0.01, *r* = 0.70 in reported cases and *r* = 0.71 in backlog separately, data obtained from Taiwan Electric Power Co., Ltd.; see Fig. 3). We use these three weather variables (maximum daily temperature, relative humidity, and wind speed) for further analysis (see Fig. 2).

Considering the lag effects of weather conditions on daily new cases of COVID-19 and the incubation period of SARS-CoV-2, the lag days of the weather were analyzed and showed a significant positive correlation between the maximum temperature and daily cases within a 14-day lag (see Table 2). To further investigate the association between the weather conditions and the number of daily cases, Fig. 2 shows the change in the correlation coefficient between the meteorological parameters and the number of daily cases within a 14-day lag. While the maximum temperature has a high correlation within 14 days with markedly higher values on lag days 6 and 10, the relative humidity and wind speed have a fairly high correlation with the number of daily cases on lag days 13 and 14, respectively. To consider the incubation period and the lag effect of weather conditions together, we assume that it was reasonable for these parameters to have a particularly high correlation on different lag days.

**Fig. 4:**
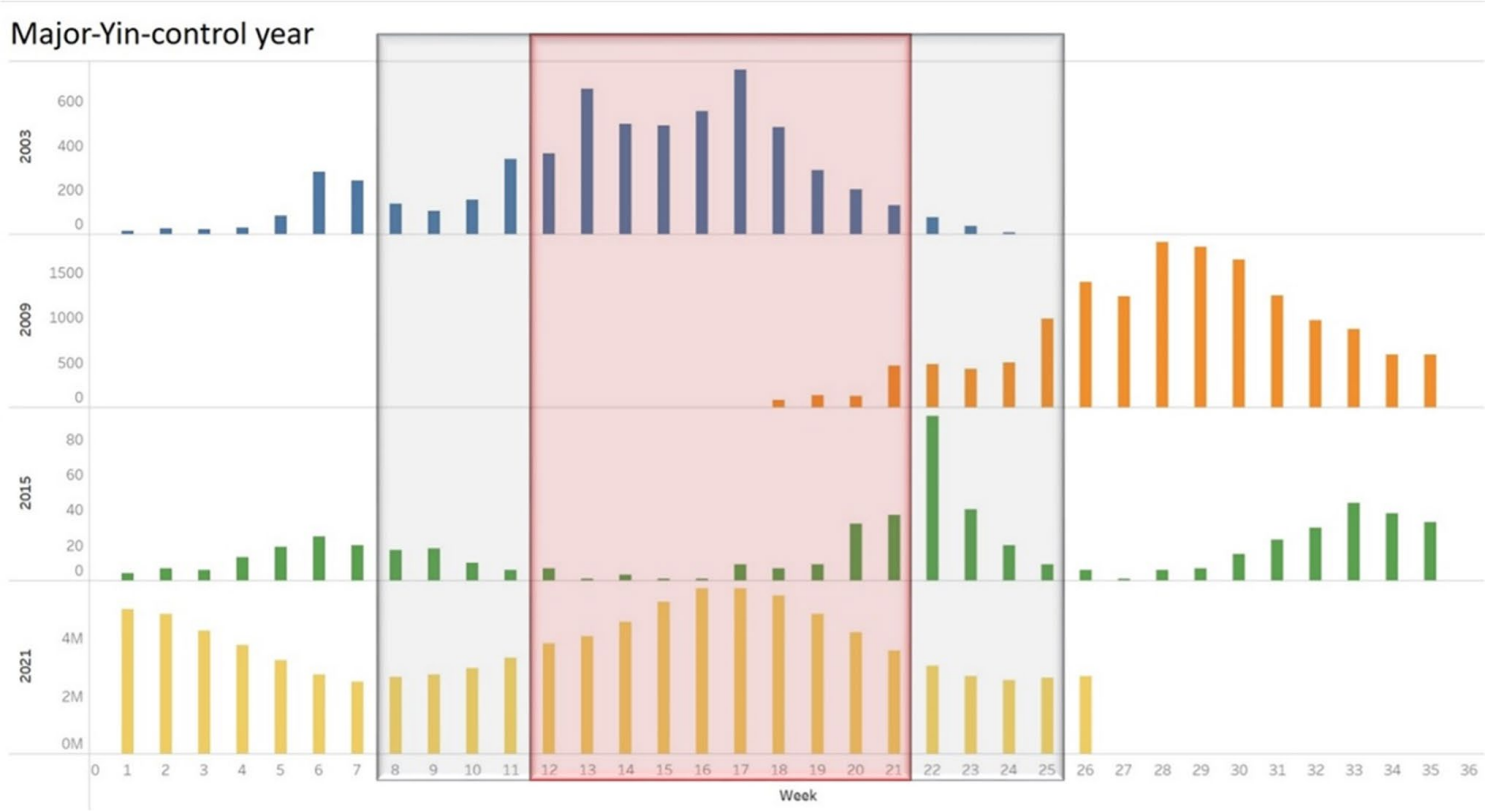
Pandemic activity in the spring of the “Major-Yin-control year” observed in 2003, 2009, 2015, and 2021

To investigate the conflicting results with other studies, we found that the second spike of daily COVID-19 cases in spring 2021 was also noticed in other countries despite the relatively high COVID-19 vaccination rate (Mathieu et al., 2021). Similarly, the SARS outbreak in 2003 also occurred in spring (Tan et al. 2005). Coincidentally, the “pandemic activity peak in spring” was noticed in the 2009 influenza A (H1N1) pandemic (Chowell et al. 2011; Tizzoni et al. 2012) and the 2015 Middle East respiratory syndrome coronavirus (MERS-CoV) outbreak in South Korea (Hsieh 2015), regardless of other outbreak peaks in another season (see Fig. 4). We speculate that there may be cyclical climate variation bringing specific weather conditions that can have an impact on infectious disease pandemic.

According to the Yunqi theory of Chinese medicine in Huangdi’s Internal Classic (Huang Di Nei Jing), some weather patterns affect the incidence of infectious disease (Zhang et al. 2016, 2015). A regular pattern of epidemic disease that occurred during the second qi (for the beginning of Spring Equinox to the beginning Grain Full, the range of March 20 to May 20 within 30 days of occurrence of deviation) was recorded every 6 years in the “Major-Yin-control year” (Unschuld PU 2011). Figure 4 shows that the peak in spring of “Major**-**Yin-control year” is observed in 2003, 2009, 2015, and 2021. In earth science, cyclical oscillations in atmospheric and geodetic signals are also observed every 5–6 years (Yu et al. 2020), which may be one of the factors affecting cyclical climate variation.

The results of this study revealed a positive significant correlation between the number of daily new confirmed cases and maximum daily temperature. The domestic outbreak of COVID-19 in Taiwan in May 2021 under UECF with an increasingly high temperature, steady high relative humidity, and slightly decreasing wind speed was observed, which affected people’s tendency in activities and encouraged the spread of the virus.

## Conclusion

We observed the COVID-19 outbreak in Taiwan in May 2021 with the maximum daily temperature significantly positively correlated with daily new COVID-19 cases, different from the trend found in the previous year. We propose that some global or regional factors such as UECF, which represents combinations of specific weather conditions, and the cyclical climate variation named the “Major-Yin-control year” may contribute to this COVID-19 outbreak in late spring. The investigation of these weather and climate patterns can help us to understand the possibility of the resurgence of outbreaks. Further analysis of weather and climate variables is essential to understand the impact of weather on the spread of infectious disease, allowing the government to make public health policies beforehand and prepare adequate supplies for the epidemic peak.

## Data Availability

All data related to this case report are documented within this manuscript.
